# Absence of altered expression of optineurin in primary open angle glaucoma patients

**Published:** 2012-06-01

**Authors:** Khaled K. Abu-Amero, Taif Anwar Azad, George L. Spaeth, Jonathan Myers, L. Jay Katz, Marlene Moster, Thomas M. Bosley

**Affiliations:** 1 Department of Ophthalmology, College of Medicine, King Saud University, Riyadh, Saudi Arabia; 2 William and Anna Goldberg Glaucoma Service, Wills Eye Hospital, Thomas Jefferson University, Philadelphia, PA; 3 Department of Ophthalmology, College of Medicine, University of Florida, Jacksonville, FL

## Abstract

**Purpose:**

To investigate the expression level of the optineurin gene (*OPTN*) in the blood of primary open angle glaucoma (POAG) patients to determine if altered expression is playing a role in primary open angle glaucoma systemically.

**Methods:**

Patients (n=47) were eligible for inclusion if they met standard clinical criteria for POAG, including age greater than 40 years, intraocular pressure ≥21 mmHg in at least one eye before treatment, normal-appearing anterior chamber angles bilaterally on gonioscopy, and optic nerve injury characteristic of POAG. Control subjects (n=27) were recruited who were free from glaucoma by examination. DNA from patient was sequenced to look for possible mutations in the coding region of *OPTN* or its promoter. RNA was extracted from leukocytes of patients and controls and converted to cDNA by reverse transcriptase enzyme, and quantitative PCR was used to assess expression levels of *OPTN* and the β-globulin gene. The ratio of *OPTN* expression to β-globulin gene expression for POAG patients was compared to that of controls and to clinical characteristics of POAG patients.

**Results:**

No mutation(s) were detected in any of the patients after sequencing the full *OPTN* gene and its promoter region. Mean *OPTN* (p≤0.35), and β-globulin (p≤0.48) gene expression values were statistically similar in POAG patients and controls. *OPTN*/β-globulin (p≤0.83) ratios were also indistinguishable between POAG patients and controls. *OPTN*/β-globulin ratios were not significantly associated with age, sex, or ethnicity of patients within the POAG group. Similarly, *OPTN*/β-globulin ratios were not significantly affected by ethnicity or clinical parameters related to POAG severity including maximum intraocular pressure, vertical cup-to-disk ratio, static perimetry mean deviation, or static perimetry pattern standard deviation.

**Conclusions:**

*OPTN* expression is not altered in the blood of POAG patients, suggesting that *OPTN* expression is not changed systemically and implying that other mechanisms are involved in POAG pathogenesis.

## Introduction

Glaucoma is one of the leading cause of blindness worldwide [1], characterized by progressive degeneration of axons in the optic nerve [2]. Primary open angle glaucoma (POAG) is the most prevalent glaucoma variant in Western countries and has risk factors that include elevated intraocular pressure (IOP) and age [3]. Elevated IOP is likely the result of the increased aqueous humor outflow resistance in the trabecular meshwork (TM) [4], but the exact mechanism and causative factors for this increase is still unclear. Up to half of all patients with POAG have a positive family history, and the risk of POAG is increased 3–9 times in first-degree relatives of POAG patients [5,6]. In addition, a maternal family history of POAG is 6–8 times more likely than a paternal family history [7-9]. These observations suggest that genetic factors may contribute to POAG, with a mitochondrial component being particularly likely [1,10-12]. A potential role of Optineurin (*OPTN*) when it was first established in 1998 as a second gene in linkage to normal tension glaucoma in the GLCE1 region at locus p15–14 on chromosome 10 [13]. Initial studies indicated that 16.7% of families with hereditary POAG had putative disease causing *OPTN* variations [14]. Subsequently, several screening studies have been conducted in different populations to identify possible changes in *OPTN* that might cause adult POAG [15-19]. *OPTN* covers a 37 kb genomic region and contains 13 exons that code for 577 amino acids. Its expression had been reported in both ocular and non-ocular tissues including the TM, non-pigmented ciliary epithelium, heart, brain, placenta, skeletal muscle, and kidney [14]. Overexpression of optineurin increases cellular protection from H_2_O_2_-induced cell death by inhibiting release of cytochrome C from mitochondria [20]. A well established mutation in *OPTN*, E50K, impairs the trafficking of *OPTN* and also is capable of causing oxidative stress to cells, possibly inducing the death of retinal ganglion cells [21]. Further recent reports suggest the role of *OPTN* in negatively regulating tumor necrosis factor-alpha (TNFα)-induced nuclear factor kappa beta (NFkB) activation [22,23].

Overexpression of *OPTN* in trabecular meshwork results in prolonged turnover rate of *OPTN* mRNA without a major impact on *OPTN* promoter activity [24].The exact role of *OPTN* in genetics remains elusive in spite of many investigations into *OPTN* gene sequence and expression variations [25]. Functional studies have also been unable to unravel its relevance to POAG [14]. Whole blood gene expression studies have been shown to have value previously in the investigation of POAG [26], other hereditary optic neuropathies [27], and diseases affecting brain anatomy and function [28,29]. The current study investigates *OPTN* expression in whole blood from POAG patients in hope that this approach will add to our knowledge of whether there is altered systemic expression of this gene in POAG.

## Methods

### Patients and controls

Patients (n=47) were evaluated in the Glaucoma Service at the Wills Eye Institute and enrolled after examination by a glaucoma specialist. Patients were eligible for inclusion if they met the following clinical criteria for POAG [30-33]: age greater than 40 years; intraocular pressure (IOP) ≥21 mmHg in one or both eyes before initiation of glaucoma treatment; normal-appearing, open anterior chamber angles bilaterally by gonioscopy; optic nerve appearance characteristic of the optic discs typically observed in primary open-angle glaucoma (with localized narrowing or absence of the neuro-retinal rim, with the amount of cupping exceeding the amount of pallor of the rim, and with asymmetric cupping of the optic discs in the two eyes); and static visual field (Humphrey Field Analyzer II; Carl Zeiss Meditec, Inc., Dublin, CA; using a full threshold 24–2 program) abnormalities typical of glaucoma (as per Advanced Glaucoma Intervention Study criteria [34]. There had to be good agreement between the appearance of the optic disc and the visual field. Exclusion criteria included historical, neuroimaging, or biochemical evidence of another possible optic neuropathic process affecting either eye, significant visual loss in both eyes not associated with glaucoma, or choosing not to participate. This research adhered to the tenets of the Declaration of Helsinki, and all patients and controls signed an informed consent approved by the Wills Eye Institute institutional review board.

All control subjects (n=27), frequently spouses of patients, had full ophthalmologic examinations and static perimetry. Each had IOPs that were <21 mmHg and symmetric in the two eyes, normal anterior chambers, optic discs that were normal and symmetric in appearance, entirely normal static perimetry OU, and no prior history of glaucoma. All controls had static perimetry performed in the same fashion as POAG patients.

### DNA testing

Five ml of peripheral blood were collected in EDTA tubes from all participating individuals. DNA was extracted using the Illustra blood genomic Prep Mini Spin Kit from GE Healthcare (Buckinghamshire, UK), and stored at −20 °C in aliquots until required.

All patient and control DNA samples were tested for mutations in the *OPTN* gene.

Successfully amplified fragments were sequenced in both directions using the M13 forward and reverse primers and the BigDye terminator v3.1 cycle sequencing kit (Applied Biosystems, Foster city, CA). Fragments were then run on the 3130*x*l Genetic Analyzer (Applied Biosystems) according to the manufacturer protocol. All the sequenced fragments were then analyzed using SeqScape software v2.6 (Applied Biosystems). Table 1 details the sequence of the primers used, the PCR annealing temperature and the expected amplicon size.

**Table 1:** Primer sequences, PCR annealing temperature and amplicon size for *OPTN*.

**Exon**	**Primer sequence**	**Annealing temp. (°C)**	**Amplicon size (bp)**
*OPTN*-1F	**TGTAAAACGACGGCCAGT**CTTGGTCGGGTGGGGTAT	57	420
*OPTN*-1R	**CAGGAAACAGCTATGACC**GCGGGTACCGTTTTCAGG	57	
*OPTN*-2F	**TGTAAAACGACGGCCAGT**TCTTAGTCTTTTCAGTATGCATTGA	59	351
*OPTN*-2R	**CAGGAAACAGCTATGACC**AGTTTATTGTGTTTTTGGTTAAGAAGA	59	
*OPTN*-3F	**TGTAAAACGACGGCCAGT**TACACACACACGCACACACA	57	253
*OPTN*-3R	**CAGGAAACAGCTATGACC**TTCCTGTGGAAAAGTCACCTG	57	
*OPTN*-4aF	**TGTAAAACGACGGCCAGT**AAGTGGGCAACTTTTGGAGT	57	391
*OPTN*-4aR	**CAGGAAACAGCTATGACC**AAGGGAGCTCACCACCAAG	57	
*OPTN*-4bF	**TGTAAAACGACGGCCAGT**ATCGCCAATGGGTTTGTG	59	376
*OPTN*-4bR	**CAGGAAACAGCTATGACC**AAGGGAGCTCACCACCAAG	59	
*OPTN*-5F	**TGTAAAACGACGGCCAGT**CAGAGCCATGTGGTCAAGTG	57	402
*OPTN*-5R	**CAGGAAACAGCTATGACC**CCATGAAAGGTTTTGATCTAGGA	57	
*OPTN*-6F	**TGTAAAACGACGGCCAGT**CCACTTCAGCCTCCCAGAG	57	382
*OPTN*-6R	**CAGGAAACAGCTATGACC**CTTGGCTTGTGTTGACAAGAA	57	
*OPTN*-7F	**TGTAAAACGACGGCCAGT**CAAAAATTCATCTTTTGTCTTTTTC	57	272
*OPTN*-7R	**CAGGAAACAGCTATGACC**ACTTCCTCAGGTCACAACATTT	57	
*OPTN*-8F	**TGTAAAACGACGGCCAGT**AAGCAGTTCCTTTAAGCTGGTC	59	352
*OPTN*-8R	**CAGGAAACAGCTATGACC**CTTTAAATGGGTGAACTGTATGG	59	
*OPTN*-9F	**TGTAAAACGACGGCCAGT**CCCAATTGTAAACAATGTTCTTTTT	57	302
*OPTN*-9R	**CAGGAAACAGCTATGACC**AAAGCAAATAACCCATCACAAGA	57	
*OPTN*-10F	**TGTAAAACGACGGCCAGT**GGCTACTAATGGTTCAGCCTGT	57	315
*OPTN*-10R	**CAGGAAACAGCTATGACC**TGTAAAAATGTATATTTCAAAGGAGGA	57	
*OPTN*-11F	**TGTAAAACGACGGCCAGT**TTATATTGTACATAACCTTGGGGTTT	59	349
*OPTN*-11R	**CAGGAAACAGCTATGACC**CAAATCCGAATTCCAATCTGTATAA	59	
*OPTN*-12F	**TGTAAAACGACGGCCAGT**CTGGAGTGTTCAGAAGGTTGG	57	293
*OPTN*-12R	**CAGGAAACAGCTATGACC**CCAGATTTAGTGAAGGATTCATGT	57	
*OPTN*-13F	**TGTAAAACGACGGCCAGT**CTAAAACAGGCAGAATTATTTCAAAAC	57	358
*OPTN*-13R	**CAGGAAACAGCTATGACC**AGAAAATTACAAACATTCTAAACACCA	57	
*OPTN*-14F	**TGTAAAACGACGGCCAGT**GGCTATTGAAGGATACAGCACT	55	330
*OPTN*-14R	**CAGGAAACAGCTATGACC**TCAAATCAGGAACGTCTTTGG	55	
*OPTN*-15F	**TGTAAAACGACGGCCAGT**TTTTTCCCCTACTTCTGTGGAC	59	279
*OPTN*-15R	**CAGGAAACAGCTATGACC**GAATCCATTGTAGAGAATGAAGTGG	59	
*OPTN*-16aF	**TGTAAAACGACGGCCAGT**GAAGTTGAACTGATGTTAAAACTCG	57	777
*OPTN*-16aR	**CAGGAAACAGCTATGACC**TAAGGATTCTACTTTGCGAGTTGATG	57	
*OPTN*-16bF	**TGTAAAACGACGGCCAGT**GAGACGACACCACTGCACTC	57	777
*OPTN*-16bR	**CAGGAAACAGCTATGACC**AACAATGTAAAAGATTCATAAGCAAAA	57	
*OPTN*-16cF	**TGTAAAACGACGGCCAGT**CCCATTCACAGTGTAAAGAAGTT	57	777
*OPTN*-16cR	**CAGGAAACAGCTATGACC**CCTCCAGTATAAGATGATAAGGGAAA	57	
*OPTN*PROM-F	**TGTAAAACGACGGCCAGT**GCAGCTTCCCTCCTCCAC	57	630
*OPTN*PROM-R	**CAGGAAACAGCTATGACC**GGGGTGCCTAGGGCTGAT	57	

F=forward; R=reverse. Bold and underlined sequences are those of the M13.

### Quantitative RT–PCR

A two-step semi-quantitative RT–PCR method was used to measure gene expression levels of *OPTN* and β-globulin in POAG patients and controls. Random hexameres were used as primers in the first step of cDNA synthesis. Total RNA (1 μg) was combined with 0.5 μg primers, 200 μM dNTPs, and sterile Milli-Q water and preheated at 65 °C for 2 min to denature secondary structures. The mixture was then cooled rapidly to 20 °C and then 10 μl 5× RT Buffer, 10 mM dithiothreitol, and 200 U Moloney Murine Leukemia Virus Reverse Transcriptase were added for a total volume of 50 μl. The RT mix was incubated at 37 °C for 90 min then stopped by heating at 95 °C for 5 min. The cDNA stock was stored at −20 °C.

Relative RT–PCR was performed to measure gene expression of *OPTN* and β-globulin according to standard guidelines [35]. Primer sequences and optimal PCR annealing temperatures (ta) are listed in Table 2. Primer sequences were designed to span intron regions to insure that no false positive PCR fragments would be generated from pseudogenes and contaminate genomic DNA. In addition, all forward PCR primers were labeled with fluorescein (6-FAM), making quantitation more accurate. Polymerase chain reactions were performed using 100 ng of cDNA, 5 pmoles of each oligonucleotide primer, 200 μM of each dNTP, 1 unit of HotStar Taq-polymerase (Qiagen, Valencia, CA) and 1× PCR buffer in a 20 μl volume. The PCR program initially started with a 95 °C denaturation for 5 min, followed by 25 cycles of 95 °C for 1 min, ta °C for 45 s, and 72 °C for 1 min. Linear amplification range for each gene was tested on the adjusted cDNA, and 25 cycles were found to be optimal for both *OPTN* and β-globulin. The PCR samples were electrophoresed on the 3130*x*l Genetic Analyzer (Applied Biosystems).

**Table 2:** Primer sequences and annealing temperature β-globulin and *OPTN* fluorescent labeled primers.

**Primer name**	**Primer sequence**	**Annealing temp. (°C)**
β-globulin F	(6-FAM)AGCCTCGCCTTTGCCGA	57
β-globulin R	CTGGTGCCTGGGGCG	
*OPTN*-LAB F	(6-FAM)GCAGGTTCCCTGGTCAGC	59
*OPTN*-LAB R	CAGGCAGCTGTTTCAAAGGT	

F=forward; R=reverse. The forward primers were labeled with 6-FAM.

### Statistical analysis

Absolute RT–PCR values were used to calculate a ratio of the *OPTN* peak area in the selected linear amplification cycle divided by that of the β-globulin gene, creating an *OPTN*/β-globulin ratio. All clinical and genetic data were analyzed using SPSS v 10 (IBM, Armonk, NY).

## Results

Age (POAG patients 67.3 years; controls 63.6 years; p≤0.17) and sex (POAG 26 males/21 females; controls 12/15; p≤0.18) of the 47 unrelated POAG patients were similar to the 27 control individuals, but ethnicity differed between the POAG group (25 Caucasian/22 African American) and the control group (23 Caucasian/4 African American; p≤0.003).

Neither POAG patients nor controls had any significant mutation(s) polymorphism(s) in the coding or promoter regions of *OPTN* after reading all sequences in the forward and reverse directions.

Mean *OPTN* (p≤0.35), and β-globulin (p≤0.48) gene expression values were statistically similar in POAG patients and controls (Table 3). *OPTN*/β-globulin (p≤0.83) ratios were also indistinguishable between POAG patients and controls. Because of the ethnic difference between the POAG group and controls, gene expression values and ratios were also compared between Caucasian POAG patients and Caucasian controls. Mean *OPTN* (p≤0.54) gene expression and *OPTN*/β-globulin (p≤0.79) ratios also did not differ between these groups.

**Table 3:** *OPTN* gene expression in POAG patients and controls.

**Parameter**	**Number POAG:Control Ψ**	**POAG**	**Control**	**p≤**
OPTN expression; mean (SD)	38:19	102205 (33682)	90885 (45922)	0.35
β-globulin expression; mean (SD)	44:24	109533 (31355)	103885 (31047)	0.48
OPTN/β-globulin; mean (SD)	38:19	1.0571 (0.606)	1.0196 (0.671)	0.83
OPTN expression in Caucasians; mean (SD)	20:18	101113 34580)	92916 (46368)	0.54
β-globulin expression in Caucasians; mean (SD)	22:20	104941 (31552)	99392 (32171)	0.58
OPTN/β-globulin in Caucasians; mean (SD)	20:18	1.1091 (0.648)	1.0512 (0.675)	0.79

POAG=primary open angle glaucoma; *OPTN*=Optineurin gene; SD=standard deviation. Ψ the number of patients or controls tested were limited to available samples and their RNA quality and quantity.

*OPTN*/β-globulin ratios were not significantly associated with age, sex, or ethnicity of patients within the POAG group (Table 4). Similarly, *OPTN*/β-globulin ratios were not significantly associated with most clinical parameters related to POAG severity, including maximum intraocular pressure, vertical cup-to-disk ratio, static perimetry mean deviation, or static perimetry pattern standard deviation. Power calculations indicated power ≤80% on these tests, leaving open the possibility of false negative type II statistical errors.

**Table 4:** Correlation between clinical parameters and *OPTN*/β-globulin ratios.

**Clinical Parameter**	***OPTN*/β-globulin**	**p≤**
Age in years	0.146	0.40
Sex	−0.131	0.45
Ethnicity	−0.120	0.49
Visual acuity [OD]	0.328	0.05
Visual acuity [OS]	0.331	0.05
Maximum IOP [OD]	−0.026	0.88
Maximum IOP [OS]	−0.099	0.57
Vertical c/d ratio [OD]	−0.065	0.71
Vertical c/d ratio [OS]	−0.091	0.60
MD [OD]	0.210	0.23
MD [OS]	0.185	0.30
PSD [OD]	−0.135	0.43
PSD [OS]	−0.157	0.37

*OPTN/*β-globulin column contains correlation coefficients; OD=right eye; OS=left eye; IOP=intraocular pressure; c/d=cup to disk; MD=Humphrey visual field mean deviation; PSD=Humphrey visual field pattern standard deviation.

## Discussion

The 47 patients reported here met rigorous clinical criteria for POAG [36] with elevated IOP, normal anterior chamber, and evidence on funduscopic exam and visual fields of glaucomatous optic nerve damage. They did not have evidence by clinical criteria of other types of glaucoma or alternative causes of optic nerve injury. None had dysmorphism or an obvious genetic syndrome. They were compared to 27 control individuals in whom POAG and other evidence of optic nerve damage were carefully excluded.

Screening the full *OPTN* gene and its promoter region revealed no mutations or significant polymorphisms in POAG patients or controls. These results are not surprising, since the prevalence of *OPTN* mutations is generally less than 5% in adult POAG populations [37]. Currently, there are 29 mutations listed in the human genome mutation database (HGMD). None of those mutation(s) were reported in the promoter region. This may indicate that the promoter region is free of mutations which could alter its expression.

Almost all studies which have investigated the *OPTN* expression in POAG have done so in cultured human TM cells [24,38-40]. However, providing conditions of TM cells capable of preserving the characteristics of TM cells in vivo is a challenge. This genetic resemblance has been exploited recently with genome-wide gene expression studies using whole blood to investigate diseases affecting brain anatomy and function [28,29,41-43]. Complex neurologic diseases such as autism [44] and schizophrenia [45] have also been investigated using similar strategies. Due to unavailability of the target tissue in glaucoma (i.e., the optic nerve), we decided to utilize whole blood from POAG patients. We studied the expression of *OPTN* gene in POAG patients and compare the rates to those of the controls. Expression of *OPTN* in blood of POAG patients was unchanged compared to that of controls (Table 3). Expression was statistically similar to that of the housekeeping gene β-globulin, and the normalized expression of *OPTN* (*OPTN*/β-globulin) also did not differ between patients and controls. *OPTN* expression did not differ between Caucasian and African American POAG patients and ethnicity matched controls.

Since our patient population had Caucasian and African Americans subjects, we compared each group of patients with their ethnicity matched controls and the results remain the same, no difference in expression level between patients and their ethnicity matched controls. Additionally, since age is a factor contributing to adult POAG, correlation between age in years and the *OPTN* expression level could not be established. Thus, aging is not a contributing factor to altered expression of this gene.

Finally, *OPTN*/β-globulin did not correlate with age or with various clinical factors associated with POAG such as visual acuity, IOP, and C/D ratio (Table 4). These findings indicate that *OPTN* expression is not altered in the blood and that other gene(s) or epigenetic factors contributing to POAG pathogenesis in this group of patients. The normal expression of *OPTN* in POAG patients contradict previous studies which showed down-regulation of *OPTN* in TM cell primary cultures and the majority of these studies had reported overexpression of *OPTN* in cultured human TM cells [38,46].

*OPTN* spans ~37-kb genomic region and contains three non-coding exons in the 5′ region and 13 exons that code for a 577-amino acid protein. Alternative splicing generates at least three different isoforms. It is possible that an imbalance in the expression of these isoforms leads to glaucoma. However, any conclusive evidence for imbalance of splice variants of *OPTN* is still lacking and remains a possible testable hypothesis [47].

Multiple interacting partners of optineurin have been reported viz huntingtin, myosin VI, rab8, and TBK1 (TANK binding kinase) [48]. Optineurin was found to be localized in golgi apparatus and upon apoptotic stimuli it translocates to the nucleus. This translocation is mediated via a GTpase rab8, an interactor of optineurin [48]. It was also found that optineurin protects the cell from oxidative damage and blocks the release of cytochrome c from mitochondria. A well documented mutation of optineurin, E50K, was found to impair the trafficking of optineurin to the nucleus and also it can cause oxidative stress to the cells which can lead to apoptosis [20]. Optineurin can mediate this action through the antiapoptotic affect of NFk-B. It is known that optineurin negatively regulates NFk-B by competing with NF-kappa-B essential modulator (NEMO), a subunit of protein kinase IKK complex involved in NFk-B regulation. I kappa B kinase (IKK) sequesters NFk-B in the cytoplasm and, upon ubiquitin-mediated destruction of IKK, it translocates to the nucleus and induces the expression of many anti-apoptotic genes. By competing with NEMO optineurin prevents the NFk-B translocation to the nucleus [49]. Upon apoptotic signal, its translocation to the nucleus can alleviate its negative regulation of NFk-B and infer a protection from cell death. But again NFk-B positively regulates optineurin [23] and upon release from apoptotic stress it can quickly render the NFk-B to its inactive state. It can be said that optineurin maintain the cell heath by its expression pattern [50]. We screened the promoter region of this gene for probable POAG causing mutations and found none. This may eliminate and support our findings that at least in the promoter region there is no apparent sequence changes which may influence expression.

Park et al. [51] has previously shown that overexpression of *OPTN* in TM cells results in prolonged turnover rate of *OPTN* mRNA but had little effect on the promoter activity [51]. The authors have speculated the interaction through control of mRNA stability. Optinuerin also interacts with metabotropic glutamate receptors (mGluR) and selectively inhibit mGluR1a [52]. Many functional studies attempted to unravel the role of *OPTN* in ocular cells but its relevance to POAG is not entirely persuasive for lack of enough genetic evidence for its major role in pathogenesis of the disease. These results contradict what we seen here of unaltered expression. This may be due to the fact that we are screening blood leucocytes and they are using TM cell lines. Providing conditions of TM cells capable of preserving the characteristics of TM cells in vivo is a challenge and this may be a weakness in studies using TM cell lines and investigating POAG pathogenesis.

In summary, we showed that *OPTN* expression is not altered in the blood of POAG patients and thus suggest other mechanism(s), gene(s) or factors contributing to the POAG pathogenesis.
